# Beovu, but not Lucentis impairs the function of the barrier formed by retinal endothelial cells in vitro

**DOI:** 10.1038/s41598-022-16770-7

**Published:** 2022-07-21

**Authors:** Heidrun L. Deissler, Catharina Busch, Armin Wolf, Matus Rehak

**Affiliations:** 1 Department of Ophthalmology, Ulm University Medical Center, Ulm, Germany; 2 Department of Ophthalmology, University Hospital Leipzig, Leipzig, Germany; 3 Department of Ophthalmology, Justus-Liebig-University Giessen, Friedrichstrasse 18, 35392 Giessen, Germany

**Keywords:** Preclinical research, Cell adhesion, Mechanisms of disease, Drug safety, Integrins, Membrane proteins, Proteins, Biochemistry, Cell biology, Drug discovery, Molecular medicine, Blotting, Electrophoresis, ELISA, Western blot, Biological techniques, Isolation, separation and purification, Eye diseases, Retinal diseases

## Abstract

Because rare, but severe adverse effects, i.e. retinal vasculitis or retinal vein occlusion, have been observed after repetitive intravitreal injections of VEGF-A-binding single-chain variable fragment brolucizumab (Beovu), we investigated its possible impact on the barrier formed by immortalized bovine retinal endothelial cells (iBREC) in comparison to that of the VEGF-A-binding Fab fragment ranibizumab (Lucentis). As a measure of stability of the barrier formed by a confluent monolayer of iBREC, we determined the cell index over seven days by continuous electric cell-substrate impedance measurements: Beovu but not Lucentis indeed significantly lowered the cell index, evident about 1.5 days after its addition, pointing to barrier impairment. Early after addition of Beovu, amounts of the integrins α5 and β1—subunits of the fibronectin receptor—had changed in opposite ways, suggesting an effect on cell adhesion due to hindered dimer formation. After exposure for eight days to Beovu, levels of claudin-1—an essential part of the iBREC barrier—were significantly lower, less claudin-1 was located at the plasma membrane after exposure to the VEGF-A antagonist for five days. Beovu did not induce secretion of inflammatory cytokines or VEGF-A. Interestingly, polysorbate-80—component of Beovu—but not polysorbate-20—in Lucentis—slightly, but significantly lowered the cell index, also associated with reduced claudin-1 expression. In summary, our results indicate that Beovu changes the behavior of retinal endothelial cells, thus providing an alternative “non-immunological” explanation for the most relevant of observed side effects.

## Introduction

Ocular diseases of high socio-economic relevance such as macular edema secondary to diabetic retinopathy or retinal vein occlusion, are associated with elevated expression of vascular endothelial growth factor-A (VEGF-A) in the vitreous, which increases the permeability of retinal endothelial cells in vivo and in vitro^1–7^. Therapeutic options targeting VEGF-A include intravitreal injections of the Fab fragment ranibizumab (Lucentis) or of brolucizumab (Beovu), a single-chain variable fragment recently approved for treatment of age-related macular degeneration and diabetic macular edema^8–11^. Both therapeutic proteins bind to all relevant splice variants of VEGF-A with high affinities^12–14^. Although these therapies are generally well tolerated, rare but severe adverse effects, i.e. retinal vasculitis or retinal vein occlusion, have been observed after repetitive intravitreal injections of Beovu^15–20^. The processes leading to these undesirable effects still remain unclear despite the potential roles of humoral and cellular immune response and particularly of specific anti-drug antibodies being broadly discussed^18–20^. In this context, it is very important to know whether Beovu itself might impair the barrier formed by the monolayer of retinal endothelial cells which are responsible for maintaining a tight inner blood-retina-barrier in vivo.

Using the well-established in vitro model of immortalized microvascular endothelial cells from the bovine retina (iBREC), developed, validated and maintained in our laboratory, we investigated whether extended exposure to Beovu, the pharmaceutical formulation of brolucizumab, harms the stable barrier formed by these cells^5,6,21^. Depending on the nature of the interfering agent, disturbances of the tight barrier formed by a confluent monolayer of primary or immortalized retinal endothelial cells of various, including human origins correlate with reduced expression or appearance at the plasma membrane of the tight junction (TJ) proteins claudin-1 and claudin-5, but can also be due to down-regulation of the fibronectin receptor subunit CD29/integrin β1 and the tetraspanin CD9/TSPAN29^4–7,22–24^. TJ-proteins together with adherens junction (AJ) proteins, e.g. vascular endothelial cadherin (VEcadherin), regulate paracellular flow, whereas the fibronectin receptor, formed by the subunits CD29/integrin β1 and CD49e/integrin α5, in complex with CD9/TSPAN29 mediates adhesion of iBREC to the extracellular matrix^25–28^. In addition to its capability to block VEGF-A-stimulated proliferation of endothelial cells (EC), brolucizumab prevents and at least transiently reverts the VEGF-A-induced dysfunction of the barrier formed by iBREC to a similar extend as ranibizumab^4,13,14,29^. To evaluate whether blocking VEGF-A-induced signal transduction per se impairs the integrity of the barrier formed by iBREC, we studied the effects of nintedanib or tivozanib which at the indicated concentrations specifically inhibit the tyrosine kinase activities of VEGF receptors (VEGFR) VEGFR1 (nintedanib: 34 nM, tivozanib: 30 nM), VEGFR2 (nintedanib: 21 nM, tivozanib: 6.5 nM) or VEGFR3 (nintedanib: 13 nM, tivozanib: 15 nM), respectively^30,31^. At concentrations of 10 nM treatment with both inhibitors indeed prevent and at least transiently revert the VEGF-A_165_-induced dysfunction of the barrier formed by an iBREC monolayer^5,6,22,32^. The VEGF-A-binding Fab fragment ranibizumab (Lucentis^®^) was included in our analyses as an already widely used reference protein not significantly affecting retinal vessels after its intravitreal application.

## Results

### Treatment with Beovu lowered the stability of the barrier formed by iBREC

**Figure 1:**
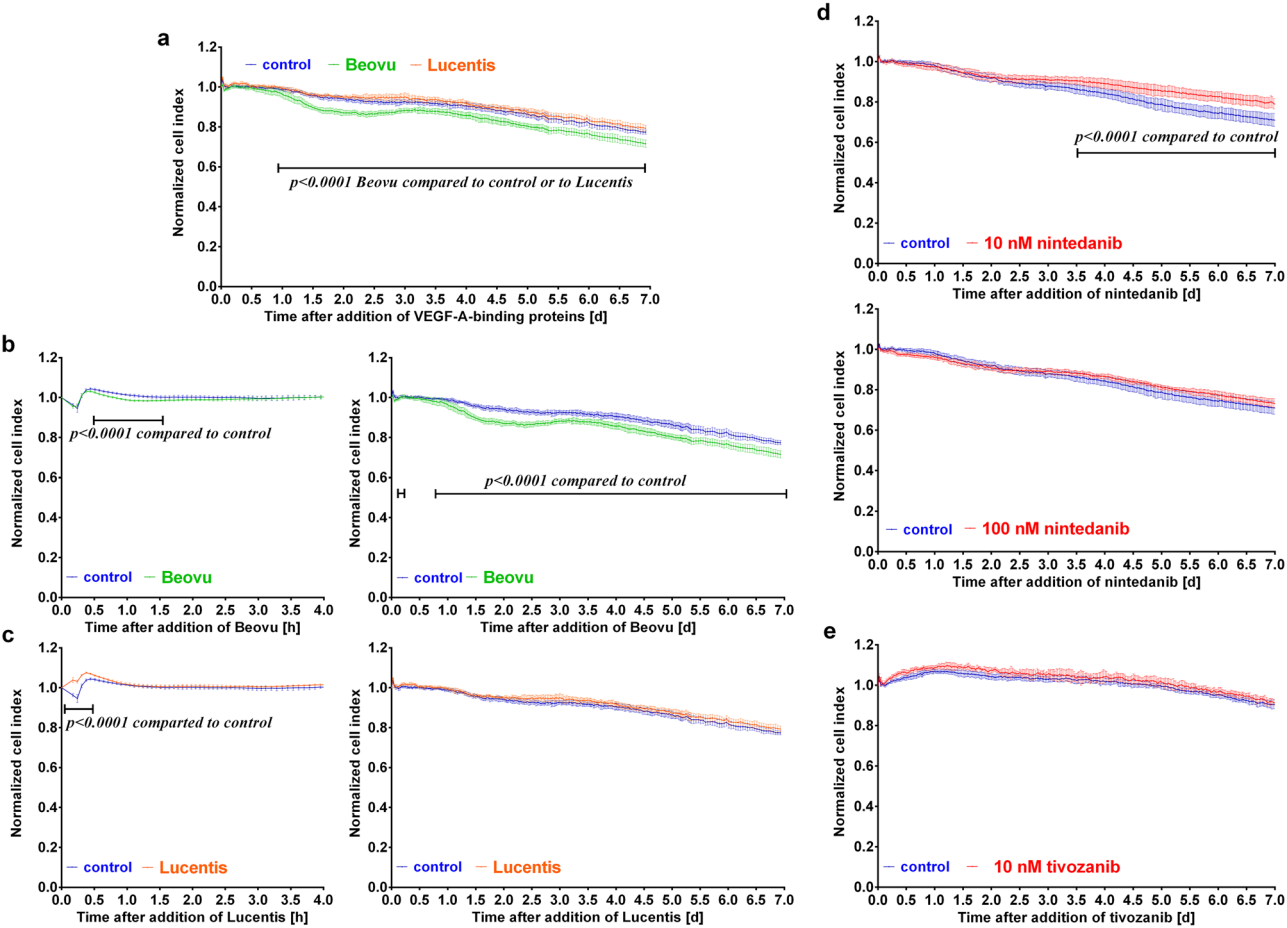
Beovu lowered the cell index of iBREC. iBREC were cultivated on gold electrodes until confluency was reached before (**a**)**,** (**b**) Beovu (*n* = 7), (**a**), (**c**) Lucentis (*n* = 6), (**d**) nintedanib (*n* = 7), (**e**) tivozanib (*n* = 3), or phosphate-buffered saline (control, blue curves; *n* ≥ 6) was added and the cell index recorded continuously as a measure of barrier function. Cell index values were normalized in relation to those measured immediately before addition of the VEGF-A antagonists and are shown as means with standard deviations. Data were analyzed with two-way ANOVA followed by Sidak’s multiple comparison test. (**a**), (**b**) Beovu significantly lowered the cell index values transiently early after its addition and persistently from ~ 1.5 days of exposure until the end of the experiment over seven days. (**a**), (**c**) Cell index values were not different in the presence of Lucentis. (**d**) Nintedanib (10 nM) significantly enhanced cell index values late after its addition, whereas the higher concentration (100 nM) had no effect. (**e**) Cell index values were also not changed by 10 nM tivozanib.

A confluent monolayer of iBREC—cultivated in cell culture medium adapted to the special needs of microvascular EC—was exposed to Beovu (final concentration: 1 mg/ml brolucizumab, Fig. 1a, b) or Lucentis (final concentration: 100 µg/ml ranibizumab, Fig. 1a, c) for up to seven days. The concentrations were chosen to correspond to those that can be reached in vivo by intravitreal injection^8,9^. As a measure of barrier stability, we determined the cell index of iBREC cultivated on gold electrodes, a method which allows the reliable detection of even subtle and transient changes: A high cell index is indicative of a tight barrier and—vice versa—an impairment of the integrity of the barrier is associated with a decline of the cell index^5^. Only Beovu (Fig. 1b, left panel), but not Lucentis (Fig. 1c, left panel), induced a slight, but significant transient decline of cell index values early after addition to the cultivated iBREC, followed by a more pronounced and sustained lowering effect, becoming evident about 1.5 days later (Fig. 1b, right panel). The extend of the Beovu-induced disturbance varied, often but not always depending on the lot of the pharmaceutical formulation. In contrast, cell index values of Lucentis-treated iBREC remained stable, reflecting those of control cells (Fig. 1c, right panel). Blocking VEGF-A_165_-induced signaling through specific inhibition of the VEGFR2 with 10 nM nintedanib (Fig. 1d, upper panel) or with 10 nM tivozanib (Fig. 1e) also did not lead to similarly low cell index values during prolonged exposure; interestingly, even slightly higher cell index values were observed with 10 nM nintedanib. Treatment of the cells with the very high concentration of 100 nM nintedanib did not change the cell index at all (Fig. 1d, lower panel).

### Beovu did not induce cellular stress or an inflammatory response

The concentrations of cytokines expressed by microvascular EC and associated with disturbance of their barrier (i.e. VEGF-A, interleukin (IL)-8 or TNFα), cellular stress (i.e. IL-6), or with an inflammatory response (i.e. IL-1β) in cell culture supernatants of iBREC treated with Beovu for two hours (→ early decline of the cell index) or thirty hours (→ persistently lowered cell index values) were measured, but their concentrations were always below the minimal amounts detectable by the ELISAs used (Table 1). Even after prolonged exposure for several days, the cells did not secrete IL-6, IL-8, TNFα or VEGF-A (Table 1).

**Table 1:** Measured cytokine concentrations in cell culture supernatants.

Target	Source of ELISA kit	Concentration ranges of standard curves (pg/ml)			Minimal detectable dose (pg/ml)	Measured concentration of target after addition of 1 mg/ml Beovu (pg/ml)								
						2 h		30 h		5 d			8 d	
						Control	Sample	Control	Sample	Control		Sample	Control	Sample
VEGF-A	CAVE00, bio-techne	0	–	1250	20	≤ 0 (*N* = 6)	≤ 0 (*N* = 6)	≤ 0 (*N* = 7)	≤ 0 (*N* = 7)	≤ 0 (*N* = 3)		≤ 0 (*N* = 3)	≤ 0 (*N* = 8)	≤ 0 (*N* = 8)
TNFα	EBTNF, Invitrogen*)	0	–	30,000	120	≤ 0 (*N* = 8)	≤ 0 (*N* = 8)	≤ 0 (*N* = 8)	≤ 0 (*N* = 8)	ND*^)^		*ND*	≤ 0 (*N* = 6)	≤ 0 (*N* = 6)
IL-1β	ESS0027, Thermo Fisher Scientific	0	–	2000	31	≤ 0 (*N* = 8)	≤ 0 (*N* = 8)	≤ 0 (*N* = 8)	≤ 0 (*N* = 8)	*ND*		*ND*	*ND*	*ND*
IL-6	ESS0029, Invitrogen	0	–	5000	78	Below minimal detectable dose								
						*(38* ± *7)* (*N* = 8)	*(42* ± *9)* (*N* = 8)	*(3* ± *5)* (*N* = 8)	*(3* ± *18)* (*N* = 8)	*(3* ± *7)* (*N* = 7)	*(11* ± *22)* (*N* = 7)		*(42* ± *52)* (*N* = 6)	*(62* ± *55)* (*N* = 6)
IL-8	ECCXCL8, Invitrogen	0	–	1500	38	≤ 0 (*N* = 8)	≤ 0 (*N* = 8)	≤ 0 (*N* = 8)	≤ 0 (*N* = 8)	*ND*		*ND*	≤ 0 (*N* = 6)	≤ 0 (*N* = 6)

*Bio-techne: Wiesbaden, Germany; Invitrogen via Thermo Fisher Scientific: Schwerte, Germany; ND: not done; means ± standard deviations are shown.

### iBREC expressed less claudin-1 after long-term treatment with Beovu

**Figure 2:**
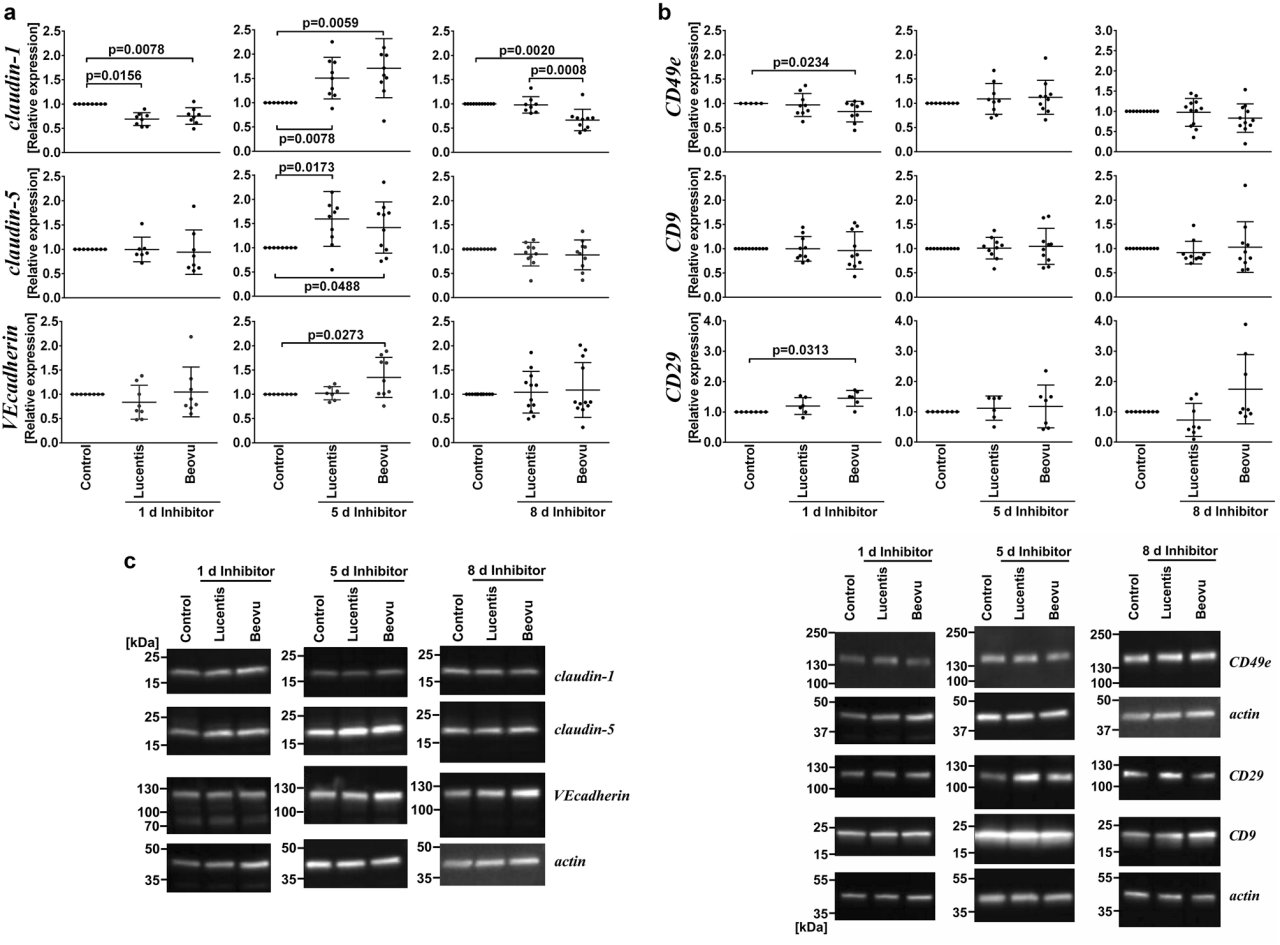
Long-term treatment with Beovu but not with Lucentis resulted in lower expression of tight junction protein claudin-1. After having reached a confluent monolayer, iBREC were exposed to Beovu or Lucentis, and cell extracts were prepared one day, five or eight days later for Western blot analyses. To compare antigen-specific signals from extracts of inhibitor-treated cells to the hypothetical value of 1.0 of normalized signals from control experiments we used the Wilcoxon signed rank test, and the Mann–Whitney U test for comparing signals from extracts of inhibitor-treated cells. Scatter blots show means and standard deviations; one dot represents the analyte-specific signal from one of multiple independent Western blot analyses. (**a**) Claudin-1 levels were similarly changed after exposure to both VEGF-A antagonists for one day or five days. After treatment with Beovu for eight days, expression of claudin-1 was significantly lower compared to control cells or Lucentis-treated iBREC. Amounts of VEcadherin were higher after exposure to Beovu for five days, those of claudin-5 after treatment with both VEGF-A inhibitors for the same period of time. (**b**) After treatment with Beovu for one day, levels of CD49e/integrin α5 were significantly lower and those of CD29/integrin β1 significantly higher compared to those of control cells. (**c**) Representative cropped images of Western blot analyses. For original images, please refer to supplemental materials (Fig. S1).

In order to better understand how exposure of iBREC to Beovu resulted in lower cell index values, we measured expression of proteins identified as determinants of barrier stability. Obvious candidate proteins that might play a role were those involved in the regulation of paracellular flow (i.e. claudin-1, claudin-5, and VEcadherin; Fig. 2a, c) and those mediating interaction of the cells with the extracellular matrix (i.e. CD9/TSPAN29, CD29/integrin β1 and CD49e/integrin α5; Fig. 2b, c). Effects of Beovu or Lucentis on their expression were analyzed after the cells had been exposed to the VEGF-A-binding proteins for one day, five or eight days. Treatment with both VEGF-A antagonists resulted in similarly changed claudin-1 expression measured one day and five days after their addition: After one day the levels were significantly lower, but significantly higher after five days compared to those of control cells (Fig. 2a, top panels). However, longer exposure (for eight days) to Beovu led to slightly, but significantly lower amounts of claudin-1 compared to those of control cells or cells similarly treated with Lucentis. Interestingly, exposure of iBREC to Beovu for five days also resulted in stronger expression of VEcadherin (Fig. 2a, bottom panels) and claudin-5 (Fig. 2a, middle panels), whereas only the expression of the latter was also higher after treatment with Beovu for the same period. In contrast, after cultivation of iBREC for only one day or as long as eight days with Beovu or Lucentis the expression levels of VEcadherin and claudin-5 were not different to those of control cells. Expression of CD49e/integrin α5 (Fig. 2b, top panels), CD9/TSPAN29 (Fig. 2b, middle panels) and CD29/integrin β1 (Fig. 2b, bottom panels) was not found to be changed in Lucentis-exposed iBREC at any time point investigated, but those of CD49e/integrin α5 were significantly lower and those of CD29/integrin β1 significantly higher in iBREC treated with Beovu for one day.

**Figure 3:**
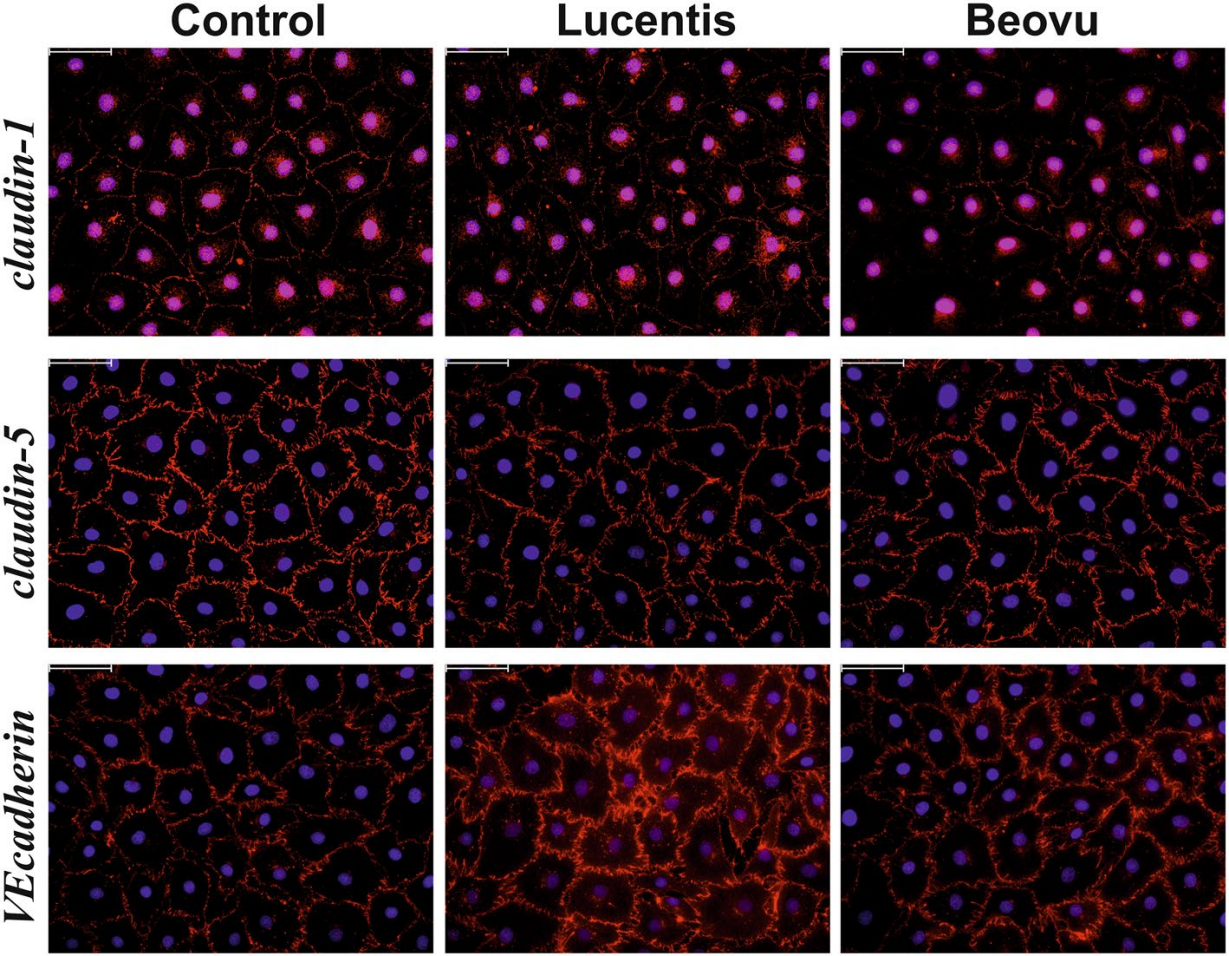
Exposure of iBREC to Beovu but not to Lucentis changed plasma membrane localization of TJ-protein claudin-1. A confluent monolayer of iBREC was treated with Beovu or Lucentis for five days before cells were fixated, and claudin-1 (upper panel), claudin-5 (middle panel) or VEcadherin (lower panel; all in red) were visualized by immunofluorescence stainings with specific antibodies. Lucentis did not markedly change the presence of any of the investigated proteins at the plasma membrane. Whereas subcellular localization of claudin-5 or VEcadherin remained largely unchanged also in the presence of Beovu, the staining specific for claudin-1 was weaker after exposure of the cells to the VEGF-A antagonist.

We also investigated by immunofluorescence stainings whether treatment of iBREC with Beovu or Lucentis for five days changed the subcellular localization of proteins involved in the regulation of paracellular flow (Fig. 3). Neither Beovu nor Lucentis markedly changed the prominent presence of VEcadherin and claudin-5 at the plasma membrane, but fractions of cells with claudin1-specific staining of the plasma membrane were significantly smaller after their exposure to Beovu (98.4 ± 2.4% for control; 95.4 ± 3.8% for Lucentis, 89.9 ± 7,7% for Beovu with p = 0.0004 compared to control and p = 0.0141 compared to Lucentis).

### Polysorbate-80 but not polysorbate-20 affected the cell index, an indicator of iBREC barrier integrity

**Figure 4:**
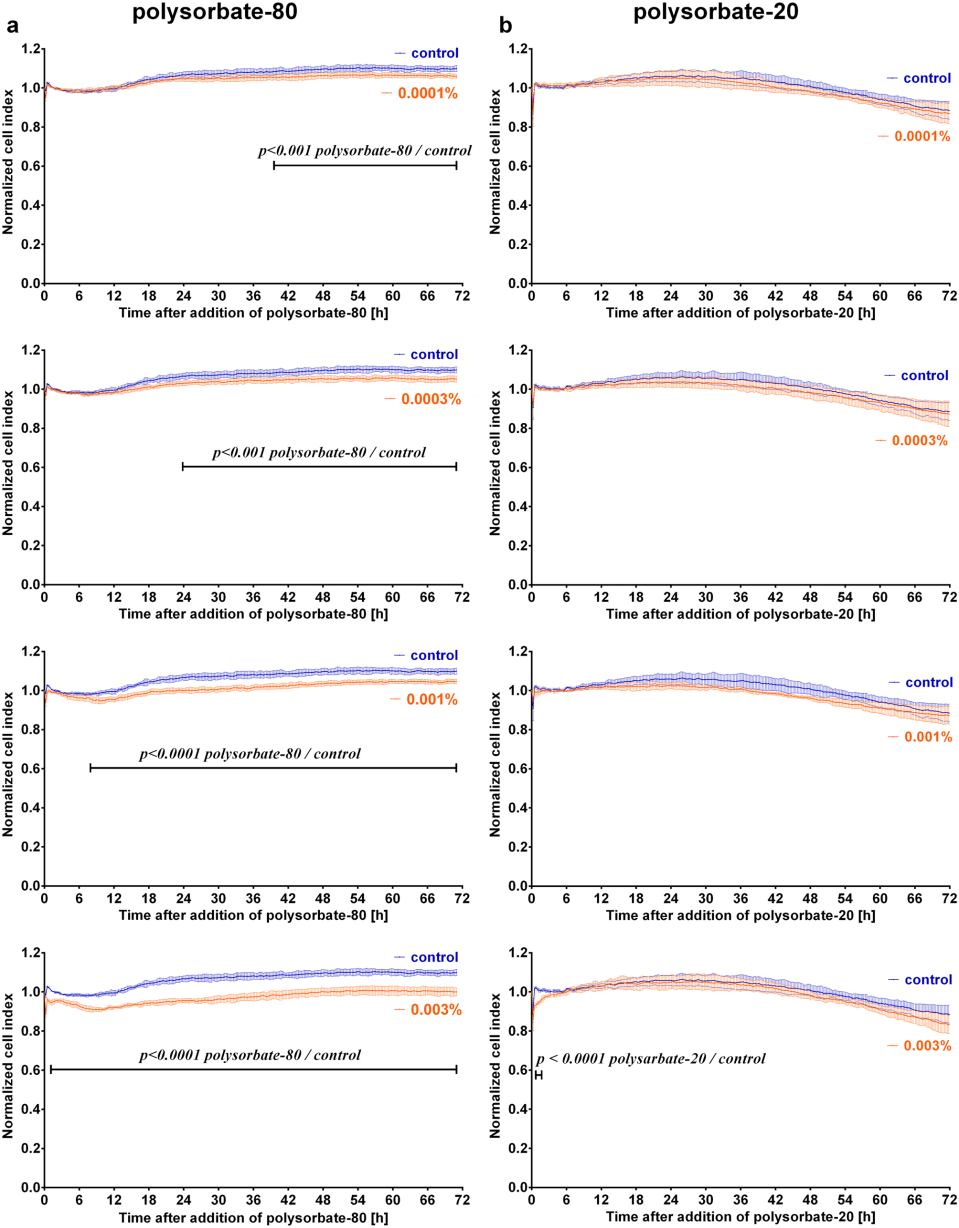
Polysorbate-80 lowered the cell index of iBREC. (**a**) Polysorbate-80 (*n* ≥ 6) or (**b**) polysorbate-20 (*n* ≥ 6) were added to a confluent monolayer of iBREC cultivated on gold electrodes and the cell index was recorded continuously as a measure of barrier function. Cell index values were normalized in relation to those measured immediately before addition of the detergents and are shown as means with standard deviations. Data were analyzed with two-way ANOVA followed by Sidak’s multiple comparison test. (**a**) Concentrations of polysorbate-80 ≥ 0.0001% significantly and persistently lowered cell index values. (**b**) Polysorbate-20 had no effect on the cell index.

**Figure 5:**
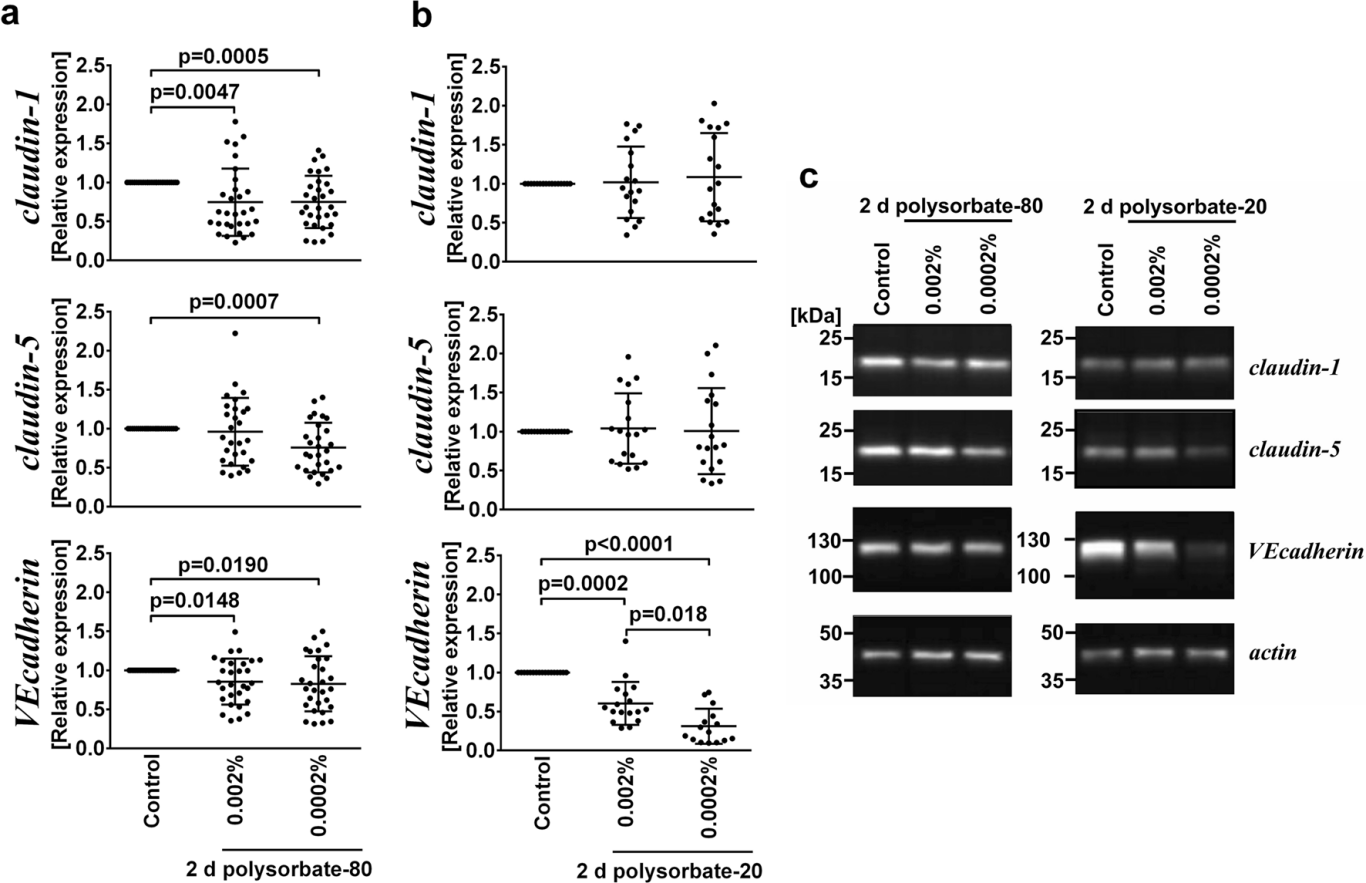
Less claudin-1 and VEcadherin were expressed after exposure of iBREC to polysorbate-80. iBREC were treated with (**a**) polysorbate-80 or (**b**) polysorbate-20 for two days before cells were harvested for preparation of cell extracts and subsequent Western blot analyses. We used the Wilcoxon signed rank test to compare antigen-specific signals from extracts of polysorbate-treated cells to the hypothetical value of 1.0 of normalized signals from control experiments and the Mann–Whitney U test for comparing signals from extracts of polysorbate-exposed cells. Scatter blots show means and standard deviations; one dot represents the analyte-specific signal from one of multiple Western blot analyses. (**a**) Levels of claudin-1 and VEcadherin were significantly lower after exposure to polysorbate-80. (**b**) Treatment with polysorbate-20 led to significantly lower expression of VEcadherin, but did not change the amount of claudin-1. (**c**) Representative cropped images of Western blot analyses (for original images see Fig. S2).

We also investigated whether components of the pharmaceutical formulation of Beovu might affect the behavior of iBREC. Polysorbate-80 and polysorbate-20—components of Beovu or Lucentis, respectively—seemed obvious candidate substances and we determined their possible effects on the cell index of an iBREC monolayer at concentrations close to 0.0002% polysorbate-80 or 0.0001% polysorbate-20 that can be reached by intravitreal injection of 50 µl of the medications. At the lowest tested concentration of 0.0001%, exposure of the cells to polysorbate-80 resulted in a slight but significant decline of the cell index visible ~ 38 h after its addition (Fig. 4a) and this effect increased considerably when higher concentrations of polysorbate-80 were applied. In contrast, in the investigated range of concentrations polysorbate-20 did not affect cell index values (Fig. 4b). Western blot analyses also revealed that significantly less claudin-1 or VEcadherin were expressed by iBREC exposed to 0.002% or 0.0002% polysorbate-80 for two days (Fig. 5a, c); levels of claudin-5 were lower only after treatment with 0.0002% polysorbate-80. Interestingly, expression of claudin-1 and claudin-5 remained stable after similar treatment of the cells with polysorbate-20, but VEcadherin was then strongly down-regulated (Fig. 5b, c).

## Discussion

To evaluate the potential relevance of an alternative, non-immunological explanation for the observed adverse effects of Beovu, we studied its impact on the retinal endothelium itself using the well-established in vitro model of immortalized bovine retinal endothelial cells (iBREC). Although of non-human origin, these cells respond to various stimuli in a very similar way as their human counterparts^21,33–36^. VEGF-A_165_ also similarly increases the permeability of primary human or bovine retinal EC and of iBREC, always accompanied by lower expression of TJ-protein claudin-1 and higher levels of the plasmalemma vesicle-associated protein^4,5,7,29,32,37,38^. Accordingly, brolucizumab (Beovu) and ranibizumab (Lucentis) prevent and revert the VEGF-A_165_-induced dysfunction of the barrier formed by iBREC^4,5,29^. This cell line is also free of contaminating cells of other types potentially interfering with characteristic behavior of the retinal EC—most importantly in this context—free of cells of the immune system^21^. iBREC were always cultivated in a culture medium adapted to the special needs of microvascular EC supplemented with fetal bovine serum ensuring optimal conditions that allow to reveal specific responses^23^. Confirming appropriate cultivation, iBREC do not secrete the marker of cellular stress IL-6 under these conditions (see Table 1)^39^. Because adverse effects of Beovu are typically observed after multiple injections when efficient inhibition or even complete blocking of the growth factor can be assumed, we decided to measure its potential effects in the absence of VEGF-A. To mimic the situation of typical patients constantly exposed to the drug, we not only assessed short-term effects but focused on a potential impact of prolonged treatment of the endothelial cells for several days.

Monitoring the cell index of iBREC cultivated on gold electrodes allows detection of subtle and transient changes of the formed barrier due to de-regulated paracellular flow and compromised adhesion of the cells^5,6,22,40,41^. iBREC showed a bi-phasic response to Beovu: A transient weak, albeit significant lowering of the cell index evident within a few hours was followed by a more pronounced and persistent decline, indicating that the barrier consisting of the cell monolayer was then less compact and likely also more penetrable. Because similar changes were not observed in analogous experiments with Lucentis, nintedanib or tivozanib, blocking of VEGF-A-induced signaling—even for a long time—seems not to be harmful to retinal EC in general. Other ocular cell types, such as cells of the retinal pigment epithelium, Muller cells or retinal ganglion cells, also tolerate inhibition of VEGF-A-induced signaling even over extended periods of time^6,42–44^. That nintedanib affected the activity of a protein kinase involved in the regulation of cellular permeability but not yet identified as a target of this inhibitor seems to be the most reasonable explanation for the observed elevated cell index during prolonged exposure of iBREC to it (Fig. 1d).

The molecular basis of the observed barrier disturbance by Beovu remains unclear, but activation of signaling pathways involved in the regulation of permeability of (retinal) endothelial cells and triggered by TNFα, IL-6, or IL-8 is rather unlikely, as Beovu-exposed iBREC did not secrete any of these cytokines^45–47^. Also, relevant amounts VEGF-A were not detected in cell culture supernatants of unchallenged or Beovu-treated iBREC, which confirms our previous finding that confluent retinal EC do not secrete this growth factor^23^. We therefore conclude that the low cell index values observed in the presence of Beovu are not caused by the action of residual bovine VEGF-A_165_ not completely inactivated by binding to the VEGF-A antagonist. Although the exact binding affinity of brolucizumab to bovine VEGF-A has not been determined, strong complex formation can be assumed due to the high homology of human and bovine VEGF-A.

Immunofluorescence stainings revealed that iBREC exposed to Beovu or Lucentis for five days still expressed a confluent monolayer and that presence at the plasma membrane of claudin-5 and VEcadherin was not affected. Results of our protein expression studies suggest a multiphasic response of the cells to Beovu: After exposure of iBREC to Beovu for one day, expression levels of the subunits of the fibronectin receptor CD49e/integrin α5 and CD29/integrin β1 had changed in opposite ways and it could well be that as a consequence of the unbalanced stoichiometry of the two subunits, less of the dimer is generated and thereby adhesion of the cells to the extracellular matrix (on the gold electrodes) is weakened^27^. Expression of low amounts of the TJ-protein claudin-1—correlating with a leaky iBREC monolayer—was observed after eight days of exposure to Beovu, also pointing to de-regulated paracellular flow^4,5^. However, the surprising observation that claudin-1 expression was found to be similarly changed by both VEGF-A antagonists one day and five days after their addition but only in the case of Lucentis normalized three days later, suggests a more complex interaction between the cells and the inhibitors. That de-regulated expression of VEcadherin was evident only in an intermediate phase of exposure for five days to Beovu supports this assumption. However, less claudin-1 located at the plasma membrane of iBREC exposed to Beovu for five days in spite of its generally increased expression might cause the observed low cell index as it obviously precedes the overall decrease of claudin-1 expression during prolonged exposure to Beovu.

The surfactants polysorbate-80 and polysobate-20, components of the formulations of Beovu or Lucentis, respectively, are ethoxylated sorbitans containing the same total number of twenty polyoxyethylene units—mostly esterified to either oleic acid or lauric acid, respectively; commercial preparations usually are mixtures also containing certain amounts of esters with other fatty acids^48^. Such surfactants improve stability of proteins and prevent formation of aggregates, but their degradation—either by residual enzymatic activity or by autooxidation—may result in precipitation of the therapeutic proteins in formulations^48–50^. Intravitreal injection of polysorbate-80 into the rabbit eye did not result in ocular inflammation, but possible disturbances of the inner blood-retina barrier might have been disregarded^51^. Therefore, it is of interest that polysorbate-80 weakly, albeit significantly, destabilized the barrier formed by iBREC even at concentrations achievable by intravitreal injection of Beovu: A significantly decreased cell index was accompanied by lower expressions of claudin-1 and VEcadherin, consistently indicating dysfunction of the barrier^4–7,25,26^. In contrast to the effects of polysorbate-80, the barrier formed by iBREC remained stable in the presence of polysorbate-20: Although less VEcadherin was then expressed, constantly high cell index values and stable expression of claudin-1 and claudin-5 were observed. These results of in vitro experiments might be of particular relevance when Beovu is injected into eyes with a relatively low volume of vitreous fluid or after repeated intravitreal injections with short intervals. Anti-drug antibodies against brolucizumab have been described and they appear to be more common than those against ranibizumab^18,20^. Intravitreally injected brolucizumab might cross the inner blood-retina barrier—possibly further promoted by the surfactant polysorbate-80—more easily, thereby leading to a higher systemic concentration and an increased risk of inducing anti-drug antibodies. These or already existing IgG cross-reacting with the VEGF-antagonist might also more likely pass the barrier to contribute to adverse reactions in the eye. Such or other interactions of the intravitreally injected drugs with systemic processes would have to be taken into consideration in investigations of possible changes of the retinal endothelium induced directly by Beovu or Lucentis based on animal models. On the other hand, an in vitro-model consisting only of retinal EC to study changes of the blood-retina barrier can be considered an over-simplification of the neurovascular unit in which other cell types also play a role^52^. Co-culture models with retinal EC, retinal pericytes and glia cells, to be used in future studies, at least bear a greater resemblance to the in vivo situation^24^.

Nevertheless, our findings clearly indicate that Beovu, but not Lucentis can harm the barrier formed by a monolayer of retinal EC in vitro. An induced dysfunction of the retinal endothelium itself might provide an additional plausible and novel explanation for the rare but severe adverse events observed after repeated intravitreal injections of Beovu.

## Methods

### Reagents, antibodies, brolucizumab and ranibizumab

Lucentis (10 mg/ml ranibizumab in 10 mM histidine-HCl, 10% α,α-trehalose dihydrate, 0.01% polysorbate-20, pH 5.5) and Beovu (120 mg/ml brolucizumab in 10 mM sodium citrate, 0.02% polysorbate-80, 5.8% sucrose, pH ~ 7.2) were purchased from Novartis Pharma GmbH (Nuremberg, Germany). Nintedanib or tivozanib (Selleckchem, Absource Diagnostics, Munich, Germany) were dissolved in dimethyl sulfoxide (#D4540, Merck, Darmstadt, Germany) to result in final solvent concentrations below 0.05% in the cell culture medium which did not affect the morphology or behavior of iBREC^5,6^. Primary and secondary antibodies used for Western blot analyses are listed with all relevant information in Table 2.

**Table 2:** Primary and secondary antibodies used.

Target	Host, type and conjugate	Source	Reducing conditions for separation	Working concentrations	Blocking solution
Actin	Mouse, monoclonal	Clone 5J11, Novus Biologicials (bio-techne) #NBP2-25,142	Both	700 ng/ml	BR*; R*
CD9	Mouse, monoclonal	Clone IVA, Exbio (Vestec, Czech Republic)	No	40 ng/ml	R
CD29	Mouse, monoclonal	Clone TS2/16 eBioscience (Thermo Fisher Scientific), #14–0299-82	No	170 ng/ml	R
CD49e	Rabbit, polyclonal	abcam (Cambridge, GB), #ab112183	Yes	0.5–1 µg/ml	BR
Claudin-1	Rabbit, polyclonal	JAY.8, Invitrogen (Thermo Fisher Scientific), #51–9000	Yes	0.25 µg/ml	BR
Claudin-5	Rabbit, polyclonal	Invitrogen (Thermo Fisher Scientific), #34–1600	Yes	100 ng/ml	BR
VEcadherin	Rabbit, polyclonal	Cell Signaling Technology B.V. (Frankfurt, Germany), #2158S	Yes	1:1,000	BR
Whole IgG, rabbit	Goat, polyclonal, coupled to HRP*^)^	Biorad (Munich, Germany), #170–5046	Both	1:30,000	BR; R
Whole IgG, mouse	Goat, polyclonal, coupled to HRP	Biorad, #170–5047	Both	1:30,000	BR; R

*BR: 1% blocking reagent (#11,096,176,001, Merck) with 0.1% Tween-20 (Biorad) in phosphate buffered saline without Ca^2+^/Mg^2+^, *R* 1% RotiBlock (#A151, Carl Roth, Karlsruhe), *HRP* horseradish peroxidase.

### Cultivation of iBREC and treatment with effectors

Experiments were performed with confluent monolayers of telomerase-immortalized microvascular EC from bovine retina (iBREC), which have been established, characterized and maintained in our laboratory^21^. iBREC were cultivated at 37 °C and 5% CO_2_ on surfaces coated with fibronectin (Corning, Amsterdam, The Netherlands or Merck, Darmstadt, Germany) in Endothelial Cell Growth Medium MV (ECGM; Promocell, Heidelberg, Germany) containing 0.4% Endothelial Cell Growth Supplement, 90 µg/ml heparin, 10 ng/ml human epidermal growth factor (hEGF), 100 nM hydrocortisone, 5% fetal bovine serum (all supplements from Promocell) and 300 µg/ml geneticin (Gibco, Thermo Fisher Scientific), as described in detail elsewhere^5,23,53^. The authenticity of the pericyte-free iBREC was regularly confirmed by analyses of the expression of proteins specific for microvascular EC.

Two days before the VEGF-A-binding protein formulations Beovu or Lucentis were added to result in final concentrations of 1 mg/ml brolucizumab or 100 µg/ml ranibizumab, respectively, the cell culture medium had been completely replaced by ECGM lacking hEGF but containing 1 µg/ml fibronectin. After one day, five or eight days of exposure, cells were harvested for preparation of whole cell extracts. Alternatively, cells were exposed to 0.0002% or 0.002% polysorbate-20 (suitable for cell culture, 53.4% lauric acid, #P2287, Merck) or polysorbate-80 (suitable for cell culture, 74.2% oleic acid, #P4780, Merck) for two days. Depending on the nature of the investigated cytokine or growth factor, cell culture supernatants were analyzed after treatment for two and 30 h, five or eight days^5,23,53^. In control experiments, cells were processed in exactly the same way only without the effector(s) investigated.

### Western blot analyses

After separation of whole cell extracts obtained from ~ 10^5^ cells by SDS polyacrylamide gel electrophoresis and transfer to a protein-binding membrane, proteins of potential relevance were determined by Western blot analyses with specific antibodies (see Table 2)^5,23,53^. Chemiluminescence signals were directly scanned with the imaging system Fusion Pulse TS (Vilbert Lourmat, VWR, Darmstadt, Germany) resulting in bright bands on dark background as described in detail elsewhere^5,23,53^. Scanned peak volumes of the protein-specific bands (more than five replicates for each condition and time point) were determined with EvolutionCapt software (Version 17.01; Vilbert Lourmat), standardized in relation to those of actin in the very same sample, and normalized to those obtained from similarly processed control cells (= 1)^5,23,53^. In the presented scatter plots, which also show means and standard deviations, data from multiple Western-blot experiments performed with several independently prepared cell extracts were pooled and each dot represents a single signal from one Western blot analysis.

### Cell index measurements

As a measure of stability of the barrier formed by an iBREC monolayer, we continuously determined the cell index of these cells cultivated on gold electrodes by electric cell-substrate impedance measurements with the microelectronic biosensor systems for cell-based assays xCELLigence RTCA DP (Acea, OLS, Bremen, Germany) as previously described^5,6,53^. Briefly, impedance was measured between gold electrodes in each individual well of an E-Plate 16 PET (Agilent, OLS) and expressed as the unit-free parameter cell index CI = (Z_i_-Z_0_)/15 Ω (RTCA Software 2.0, Acea) with Z_i_ being the impedance measured at an individual time point and Z_0_ the impedance read at the start of the experiment^5,54^. iBREC (~ 10^4^ cells) were placed in a fibronectin-coated well and cultivated until a high and constant cell index indicative of a confluent monolayer (CI ~ 18; cell index measurements every 15 min) was reached three to four days later. After complete replacement of the cell culture medium with ECGM lacking hEGF but containing 1 µg/ml fibronectin, the cell index was measured every 15 min for two days, before Beovu (final concentration of brolucizumab: 1 mg/ml), Lucentis (final concentration of ranibizumab: 100 µg/ml), nintedanib or tivozanib (final concentrations of both inhibitors: 10 nM or 100 nM) were added. The cell index was then determined every two minutes for 90 min, every five minutes for 62 h, and every 15 min until the end of the experiments. Alternatively, polysorbate-20 or polysorbate-80 was added (final concentrations: 0.0001%, 0.0003%, 0.001% or 0.003%) to confluent iBREC cultivated in ECGM lacking hEGF but containing 1 µg/ml fibronectin for two days, and the cell index was monitored over three days as described above. All experiments were performed at least three times and data were then obtained from three to eight individual wells for each condition and time point, and recorded cell index values were normalized in relation to those measured immediately before addition of the inhibitors (RTCA Software Pro 2.3.4 (Basic), Agilent). The results were analyzed and converted to graphs showing means and standard deviations with Graph Pad Prism 6 (Graph Pad Software, San Diego, USA)^5,23,53^.

### Immunofluorescence staining

After exposing confluent monolayers of iBREC—cultivated on fibronectin-coated two-chamber slides (x-well PCA Tissue Culture Chambers; Sarstedt, Nuembrecht, Germany)—to Beovu or Lucentis for five days as described above, cells were fixated in methanol for 7.5 min at -20 °C. Slides were blocked in 10% ImmunoBlock (Roth)/phosphate-buffered saline without Ca^2+^ and Mg^2+^ (PBSd) for one hour before they were incubated with specific antibodies against claudin-1 (rabbit polyclonal; Aviva Systems Biology via Biozol, Eching, Germany, #ARP33623_P50; 4 µg/ml), claudin-5 (see Table 2; 2.5 µg/ml) or VEcadherin (see Table 2; 1:100) for one hour and subsequently with goat F(ab’)_2_ fragments (coupled to AlexaFluor595) directed against rabbit IgG, H + L chains (#A11072, Invitrogen, Thermo Fisher Scientific; 1:500) for 30 min; all antibodies were diluted in 1% Immunoblock/PBSd. Then cells were embedded in ProLong Gold Antifade Mountant (Thermo Fisher Scientific) which contains 4’,6-diamidino-2-phenylindole (DAPI) to stain the nuclei. Cells were examined by fluorescence microscopy (DM4000B, LAS X, Leica, Wetzlar, Germany) and portions of cells with claudin-1-specific plasma membrane staining were counted in at least twelve randomly chosen microscopic fields containing ~ 30 cells/field^5,23,29^.

### Determination of cytokines by ELISA

The concentrations of VEGF-A, TNFα, IL-1β, IL-6 or IL-8 were determined in undiluted cell culture supernatants of iBREC treated with Beovu by ELISA, using the kits listed in Table 1^23,54^. We processed triplicate or quadruplicate samples according to the manufacturers’ instructions and measured the analyte-dependent absorbance at 450 nm (reference wavelength: 570 nm) 10–20 min after addition of the stop solution with an Infinite 200Pro spectrophotometer controlled by Tecan i software (Tecan, Crailsheim, Germany); standard curves were always generated in parallel to the analyses of samples^54^.

### Statistical analyses

We used the Wilcoxon signed rank test to compare antigen-specific Western blot signals from effector-treated cells to the hypothetical value of 1.00 of normalized signals from control cells, and the Mann–Whitney U test to compare those of differently treated cells. The Wilcoxon signed rank test makes allowances for the variability of the values obtained from controls although they appear without standard deviations (SD = 0). To analyze data from cell index measurements, the two-way ANOVA followed by Sidak’s multiple comparison test was used. Differences resulting in p-values below 0.05 were considered significant. All statistical analyses were performed with Graph Pad Prism 6; means and standard deviations are provided as numbers, graphs or in scatter plots.

### Ethical approval

The authors have no ethical conflicts to disclose. This article does not contain any results of studies with human participants or animals.

## Supplementary Information

Supplementary Information.

## Data Availability

The original data used to support the findings of this study are either included in the manuscript or are available from the corresponding author upon request.

## References

[1] Aiello LP (1994). Vascular endothelial growth factor in ocular fluid of patients with diabetic retinopathy and other retinal disorders. N. Engl. J. Med..

[2] Antonetti DA, Barber AJ, Hollinger LA, Wolpert EB, Gardner TW (1999). Vascular endothelial growth factor induces rapid phosphorylation of tight junction proteins occludin and zonula occludens 1. J. Biol. Chem..

[3] Qaum T (2001). VEGF-initiated blood-retinal barrier breakdown in early diabetes. Invest. Ophthalmol. Vis. Sci..

[4] Deissler H, Deissler H, Lang GE (2011). Inhibition of VEGF is sufficient to completely restore barrier malfunction induced by growth factors in microvascular retinal endothelial cells. Br. J. Ophthalmol..

[5] Deissler HL, Lang GK, Lang GE (2017). Inhibition of single routes of intracellular signaling is not sufficient to neutralize the biphasic disturbance of a retinal endothelial cell barrier induced by VEGF-A_165_. Cell. Physiol. Biochem..

[6] Deissler HL (2020). VEGF receptor 2 inhibitor nintedanib completely reverts VEGF-A_165_-induced disturbances of barriers formed by retinal endothelial cells or long-term cultivated ARPE-19 cells. Exp. Eye Res..

[7] Suarez S (2014). Modulation of VEGF-induced retinal vascular permeability by peroxisome proliferator-activated receptor-β/δ. Invest. Ophthalmol. Vis. Sci..

[8] Lang GE (2013). Two-year safety and efficacy of ranibizumab 0.5 mg in diabetic macular edema: interim analysis of the RESTORE extension study. Ophthalmology.

[9] Dugel PU (2020). HAWK and HARRIER: Phase 3, multicenter, randomized, double-masked trials of brolucizumab for neovascular age-related macular degeneration. Ophthalmology.

[10] Nguyen QD (2020). Brolucizumab: Evolution through preclinical and clinical studies and the implications for the management of neovascular age-related macular degeneration. Ophthalmology.

[11] Brown DM (2022). KESTREL and KITE: 52-Week results from two phase III pivotal trials of brolucizumab for diabetic macular edema. Am. J. Ophthalmol..

[12] Ferrara N, Damico L, Shams N, Lowman H, Kim R (2006). Development of ranibizumab, an anti-vascular endothelial growth factor antigen binding fragment, as therapy for neovascular age-related macular degeneration. Retina.

[13] Gaudreault J (2012). Preclinical pharmacology and safety of ESBA1008, a single-chain antibody fragment, investigated as potential treatment for age related macular degeneration. Invest. Ophthalmol. Vis. Sci..

[14] Tietz J (2015). Affinity and potency of RTH258 (ESBA1008), a novel inhibitor of vascular endothelial growth factor A for the treatment of retinal disorders. Invest. Ophthalmol. Vis. Sci..

[15] Baumal CR (2020). Retinal vasculitis and intraocular inflammation after intravitreal injection of brolucizumab. Ophthalmology.

[16] Witkin AJ (2020). Occlusive retinal vasculitis following intravitreal brolucizumab. J. Vitreoretin. Dis..

[17] Singer M (2021). Clinical characteristics and outcomes of eyes with intraocular inflammation after brolucizumab: Post hoc analysis of HAWK and HARRIER. Ophthalmol. Retina.

[18] Sharma A (2021). Understanding retinal vasculitis associated with brolucizumab: complex pathophysiology or Occam’s razor?. Ocul. Immunol. Inflamm..

[19] Anderson WJ (2021). Mechanisms of sterile inflammation after intravitreal injection of antiangiogenic drugs: a narrative review. Int. J. Retina Vitreous.

[20] Busch M (2022). Anti-drug antibodies to brolucizumab and ranibizumab in serum and vitreous of patients with ocular disease. Acta Ophthalmol..

[21] Deissler H, Deissler H, Lang GK, Lang GE (2005). Generation and characterization of iBREC: novel hTERT-immortalized bovine retinal endothelial cells. Int. J. Mol. Med..

[22] Jäckle A (2020). Sitagliptin and the blood-retina barrier: Effects on retinal endothelial cells manifested only after prolonged exposure. J. Diab. Res..

[23] Busch C (2021). Type of culture medium determines properties of cultivated retinal endothelial cells: induction of substantial phenotypic conversion by standard DMEM. Heliyon.

[24] Wisniewska-Kruk J (2012). A novel co-culture model of the blood-retinal barrier based on primary retinal endothelial cells, pericytes and astrocytes. Exp. Eye Res..

[25] Dejana E, Tournier-Lasserve E, Weinstein BM (2009). The control of vascular integrity by endothelial cell junctions: molecular basis and pathological implications. Dev. Cell..

[26] Jeong JH, Nguyen HK, Lee JE, Suh W (2016). Therapeutic effect of apatinib-loaded nanoparticles on diabetes-induced retinal vascular leakage. Int. J. Nanomed..

[27] Akiyama SK (1996). Integrins in cell adhesion and signaling. Hum. Cell..

[28] Deissler H, Kuhn EM, Lang GE, Deissler H (2007). Tetraspanin CD9 is involved in the migration of retinal microvascular endothelial cells. Int. J. Mol. Med..

[29] Deissler HL, Rehak M, Busch C, Wolf A (2022). Blocking of VEGF-A is not sufficient to completely revert its long-term effects on the barrier formed by retinal endothelial cells. Exp. Eye Res..

[30] Hilberg F (2008). BIBF1120: Triple angiokinase inhibitor with sustained receptor blockade and good antitumor efficacy. Cancer Res..

[31] Nakamura K (2006). KRN951, a highly potent inhibitor of vascular endothelial growth factor receptor tyrosine kinases, has antitumor activities and affects functional vascular properties. Cancer Res..

[32] Deissler HL, Rehak M, Wolf A (2022). Impairment of the retinal endothelial cell barrier induced by long-term treatment with VEGF-A_165_ no longer depends on the growth factor’s presence. Biomolecules.

[33] Castellon R (2002). Effects of angiogenic growth factor combinations on retinal endothelial cells. Exp. Eye Res..

[34] Deissler H, Deissler H, Lang S, Lang GE (2008). VEGF-induced effects on proliferation, migration and tight junctions are restored by ranibizumab (Lucentis) in microvascular retinal endothelial cells. Br. J. Ophthalmol..

[35] Stewart EA, Samaranayake GJ, Browning AC, Hopkinson A, Amoaku WM (2011). Comparison of choroidal and retinal endothelial cells: Characteristics and response to VEGF-isoforms and anti-VEGF treatment. Exp. Eye Res..

[36] Deissler HL, Deissler H, Lang GK, Lang GE (2013). Ranibizumab efficiently blocks migration but not proliferation induced by growth factor combinations including VEGF in retinal endothelial cells. Graefes Arch. Clin. Exp. Ophthalmol..

[37] Wisniewska-Kruk J (2016). Plasmalemma vesicle-associated protein has a key role in blood-retinal barrier loss. Am. J. Pathol..

[38] Haque N (2021). VEGFA165 activation of PLVAP expression utilizes the p38-MAPK and AKT signaling pathways in primary human retinal endothelial Cells. Invest. Ophthalmol. Vis. Sci..

[39] Strohl LL (2013). Norepinephrine and adenosine-5'-triphosphate synergize in inducing IL-6 production by human dermal microvascular endothelial cells. Cytokine.

[40] Atienza JM, Zhu J, Wang X, Xu X, Abassi Y (2005). Dynamic monitoring of cell adhesion and spreading on microelectronic sensor arrays. J. Biomol. Screen..

[41] Bischoff I (2016). Pitfalls in assessing microvascular endothelial barrier function: impedance-based devices versus the classic macromolecular tracer assay. Sci. Rep..

[42] Saint-Geniez M (2008). Endogenous VEGF is required for visual function: evidence for a survival role on Müller cells and photoreceptors. PLoS ONE.

[43] Schottler J (2018). Long-term treatment with anti-VEGF does not induce cell aging in primary retinal pigment epithelium. Exp. Eye Res..

[44] Miki A (2010). Prolonged blockade of VEGF receptors does not damage retinal photoreceptors or ganglion cells. J. Cell. Physiol..

[45] Aveleira CA, Lin CM, Abcouwer SF, Ambrósio AF, Antonetti DA (2010). TNF-α signals through PKCζ/NF-κB to alter the tight junction complex and increase retinal endothelial cell permeability. Diabetes.

[46] Valle ML (2019). Inhibition of interleukin-6 trans-signaling prevents inflammation and endothelial barrier disruption in retinal endothelial cells. Exp. Eye Res..

[47] Yu H (2013). Interleukin-8 regulates endothelial permeability by down-regulation of tight junction but not dependent on integrins induced focal adhesions. Int. J. Biol. Sci..

[48] Martos A (2017). Trends on analytical characterization of polysorbates and their degradation products in biopharmaceutical formulations. J. Pharm. Sci..

[49] Borisov OV, Ji JA, Wang YJ (2015). Oxidative degradation of polysorbate surfactants studied by liquid chromatography-mass spectrometry. J. Pharm. Sci..

[50] Zhang S, Xiao H, Li N (2021). Degradation of polysorbate 20 by sialate O-acetylesterase in monoclonal antibody formulations. J. Pharm. Sci..

[51] Damico FM (2017). Intravitreal injection of polysorbate 80: a functional and morphological study. Rev. Col. Bras. Cir..

[52] Klaassen I (2013). Molecular basis of the inner blood-retinal barrier and its breakdown in diabetic macular edema and other pathological conditions. Prog. Retin. Eye Res..

[53] Deissler HL, Sommer K, Lang GK, Lang GE (2020). Transport and fate of aflibercept in VEGF-A_165_-challenged retinal endothelial cells. Exp. Eye Res..

[54] Sun M (2012). A dynamic real-time method for monitoring epithelial barrier function in vitro. Anal. Biochem..

