# Supplementary material for: Beovu, but not Lucentis impairs the function of the barrier formed by retinal endothelial cells in vitro
Source: Sci Rep. 2022 Jul 21;12:12493. doi: 10.1038/s41598-022-16770-7 (PMC9304347; doi:10.1038/s41598-022-16770-7)

Supplementary material to:
Beovu®, but not Lucentis® impairs the function of the barrier formed by retinal endothelial cells *in vitro*

Heidrun L. Deissler, Catharina Busch, Armin Wolf, Matus Rehak

Original images of Western-blot analyses:

To determine presence of multiple antigens in parallel, the protein-bound membrane was cut in two or three pieces (see following pages) and exposed to the mentioned antibodies. Afterwards the membrane parts were incubated for 45 min in Restore Plus Western Blot Stripping Buffer (Thermo Fisher Scientific, #46430) at room temperature to remove bound antibodies. After washing three times for 5 min with 0.1% Tween-20/PBSd and blocking, the membrane parts were exposed to another set of antibodies. This procedure was followed for a second time, if necessary.

Chemiluminescence signals were always directly scanned with the imaging system Fusion Pulse TS (Vilbert Lourmat), a black-and-white image of the membrane containing the prestained size marker proteins (peqGOLD Protein Marker V, VWR, avantor) was taken in parallel. The inverse image of the antibody-specific signal (black bands on grey background) was merged with the corresponding image of the membrane containing the prestained size marker with EvolutionCapt software (Version 17.01; Vilbert Lourmat). Labels were added to the merged image using the editing-function of the software.

Supplementary Figure S1: Original images of Fig. 2

1. Cell extracts were prepared of iBREC exposed to Beovu or Lucentis for 1 d.
2. Proteins were separated by SDS-PAGE under reducing conditions. After protein transfer, the membrane was cut into three pieces below 35 kDa und above 55 kDa (see arrows). After blocking, the upper part was exposed to antibodies against VE cadherin, the middle part against actin (antibody binds unspecifically to proteins of PeqGold Protein Marker V (avantor), the lower half to claudin-1-specific antibodies:

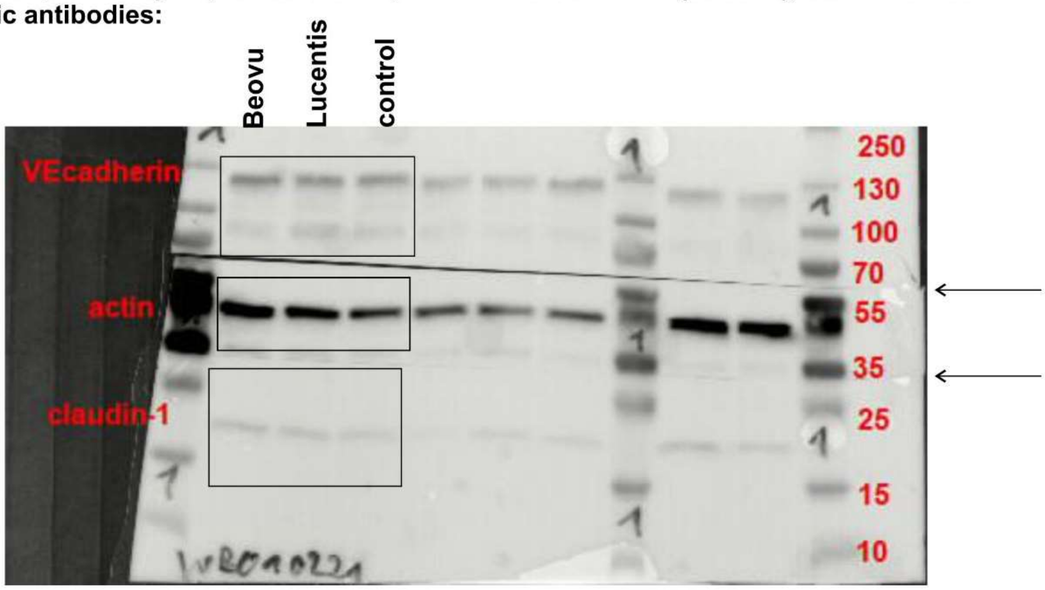

3. Lower part of the membrane was washed in Restore Plus Western Blot Stripping Buffer at RT for 45 min to remove bound antibodies. After blocking, it was exposed to claudin-5-specific antibodies:

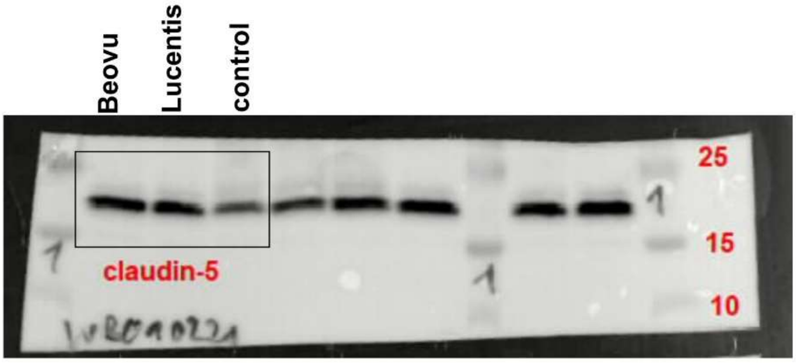

4. Upper part of the membrane was also washed in Restore Plus Western Blot Stripping Buffer at RT for 45 min to remove bound antibodies. After blocking, it was exposed to CD49e-specific antibodies:

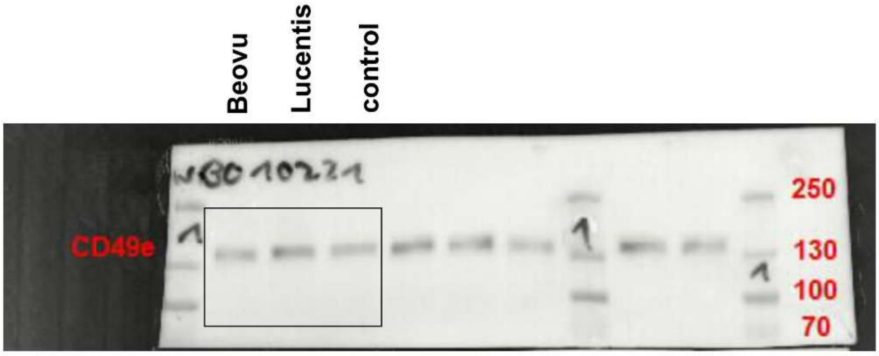

1. Cell extracts were prepared from iBREC exposed to Beovu or Lucentis for **5 d**.
2. Proteins were separated by SDS-PAGE under reducing conditions. After protein transfer, the membrane was cut into two halves just above 70 kDa (see arrow). After blocking, the upper half was exposed to antibodies against VE cadherin, the lower half to claudin-1-specific antibodies:

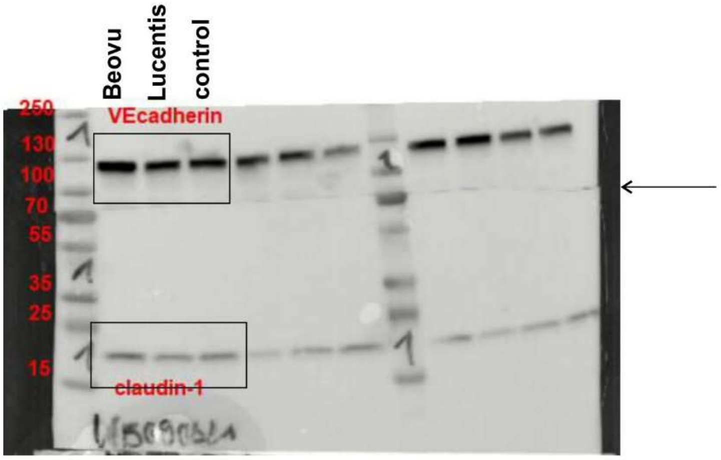

3. Lower part of the membrane was washed in Restore Plus Western Blot Stripping Buffer at RT for 45 min to remove bound antibodies. After blocking, it was exposed to antibodies binding to actin (antibody binds unspecifically to proteins of PeqGold Protein Marker V (avantor):

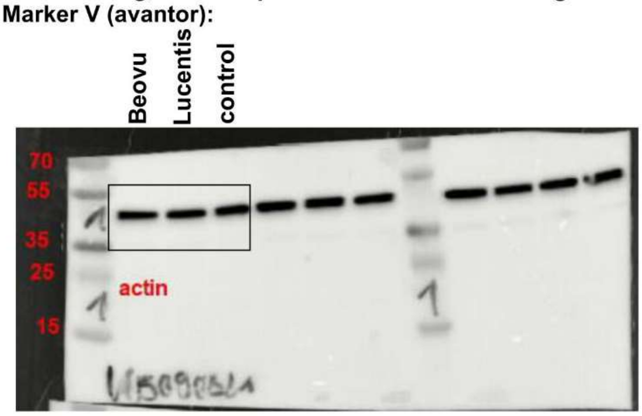

4. Lower part of the membrane was washed in Restore Plus Western Blot Stripping Buffer for a 2nd time at RT for 45 min to remove bound antibodies. After blocking, it was exposed to claudin-5-specific antibodies:

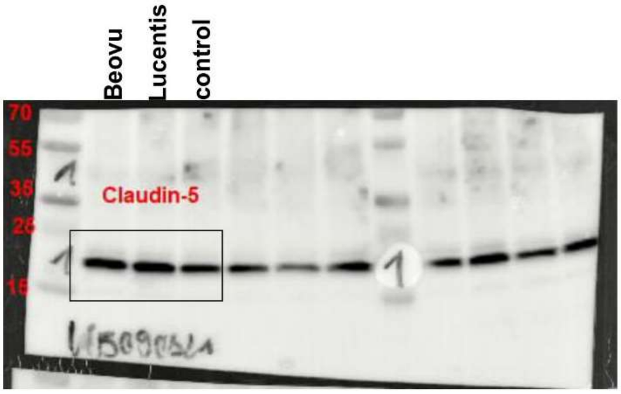

5. Upper part of the membrane was also washed in Restore Plus Western Blot Stripping Buffer at RT for 45 min to remove bound antibodies. After blocking, it was exposed to CD49e-specific antibodies:

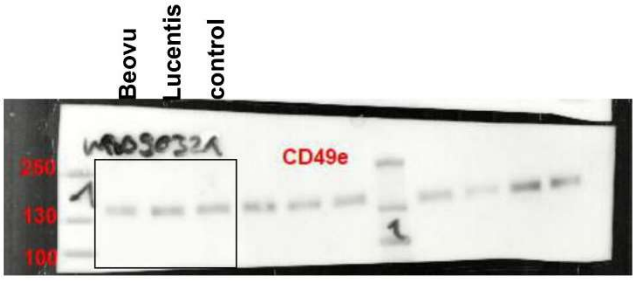

1. Cell extracts were prepared from iBREC exposed to Beovu or Lucentis for **8 d**.

2. Proteins were separated by SDS-PAGE under reducing conditions. After protein transfer, the membrane was cut into two halves just above 70 kDa (see arrow). After blocking, the upper half was exposed to antibodies against VE cadherin, the lower half to claudin-1-specific antibodies:

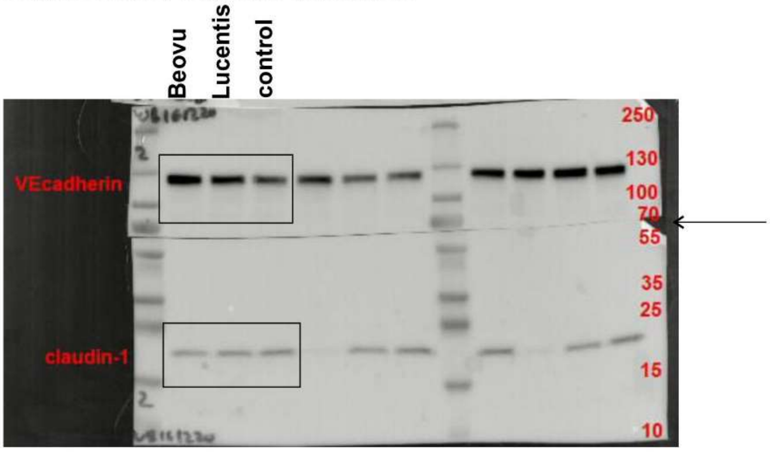

3. Lower part of the membrane was washed in Restore Plus Western Blot Stripping Buffer at RT for 45 min to remove bound antibodies. After blocking, it was exposed to antibodies binding to actin:

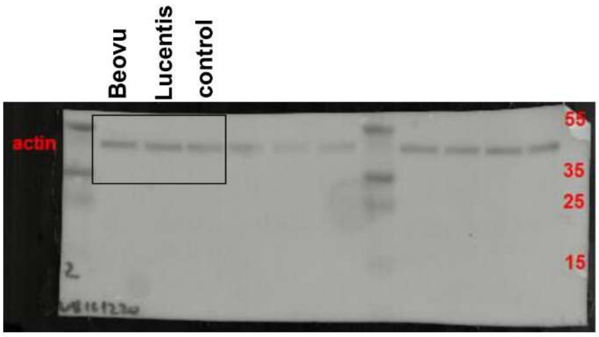

4. Lower part of the membrane was washed in Restore Plus Western Blot Stripping Buffer for a 2nd time at RT for 45 min to remove bound antibodies. After blocking, it was exposed to claudin-5-specific antibodies:

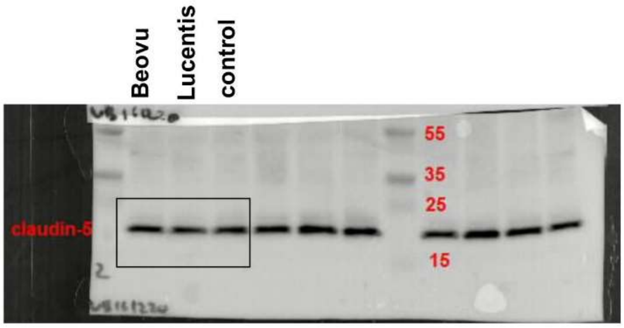

5. Upper part of the membrane was also washed in Restore Plus Western Blot Stripping Buffer at RT for 45 min to remove bound antibodies. After blocking, it was exposed to CD49e-specific antibodies:

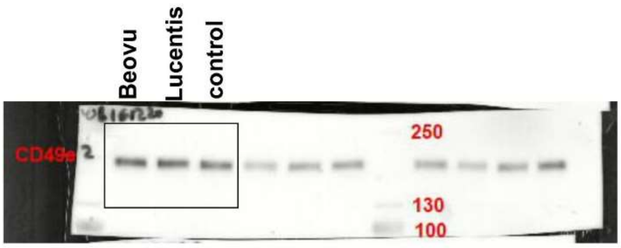

1. Cell extracts were prepared from iBREC exposed to Beovu or Lucentis for 1 d.
2. Proteins were separated by SDS-PAGE under non-reducing conditions. After protein transfer, membrane was cut into two halves just above 70 kDa (see arrow). After blocking, the upper part was exposed to antibodies against CD29, the lower part against actin (antibody binds unspecifically to proteins of PeqGold Protein Marker V (avantor):

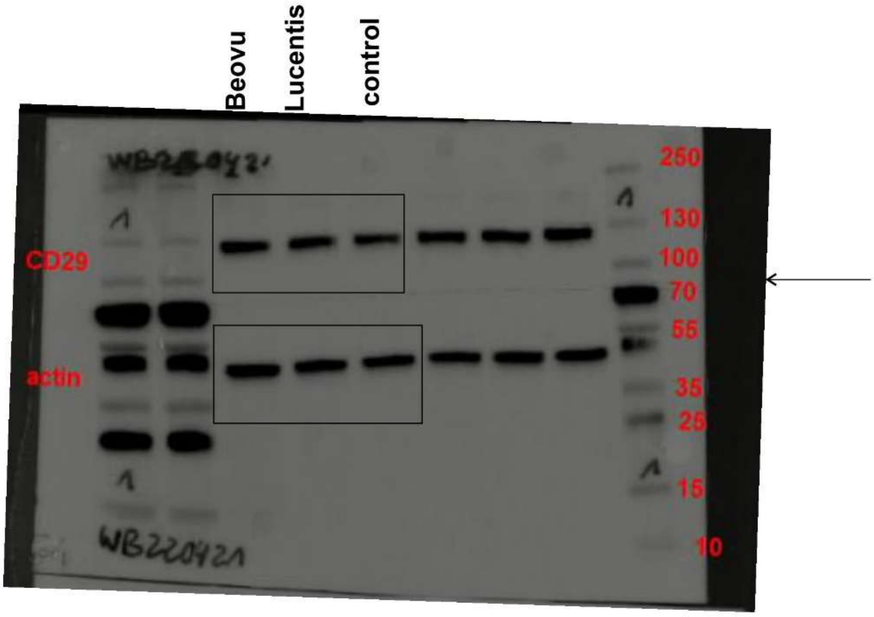

3. Lower part of the membrane was washed in Restore Plus Western Blot Stripping Buffer at RT for 45 min to remove bound antibodies. After blocking, it was exposed to CD9-specific antibodies:

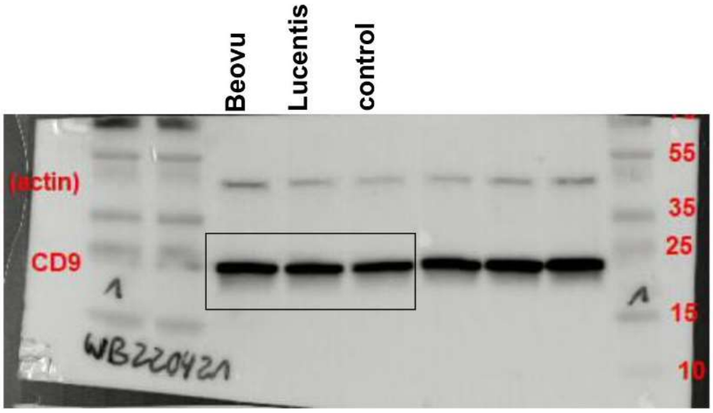

1. Cell extracts were prepared from iBREC exposed to Beovu or Lucentis for 5 d.
2. Proteins were separated by SDS-PAGE under non-reducing conditions. After protein transfer, the membrane was cut into two halves just below 70 kDa (see arrow). After blocking, the upper part was exposed to antibodies against CD29, the lower part against VEGFA-specific antibodies:

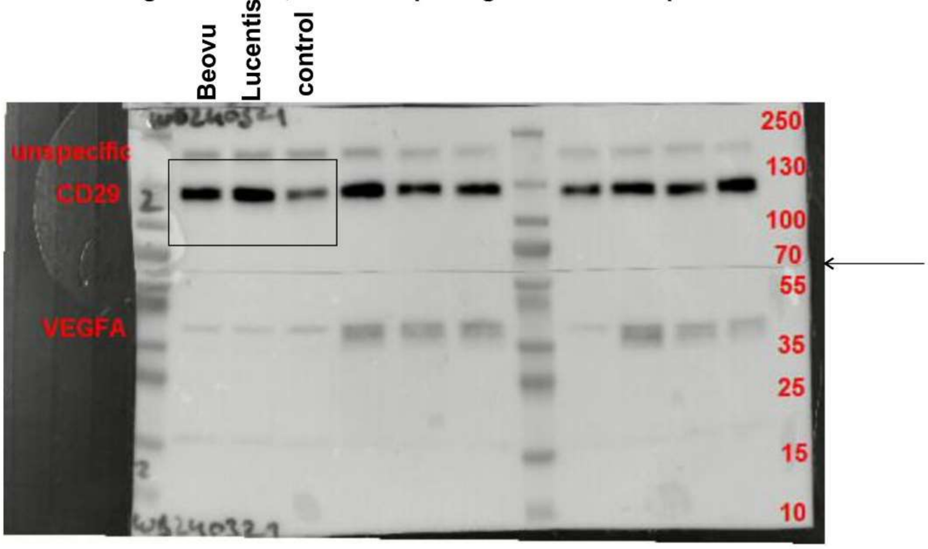

3. Lower part of the membrane was washed in Restore Plus Western Blot Stripping Buffer at RT for 45 min to remove bound antibodies. After blocking, it was exposed to antibodies against actin (antibody binds unspecifically to proteins of PeqGold Protein Marker V (avantor):

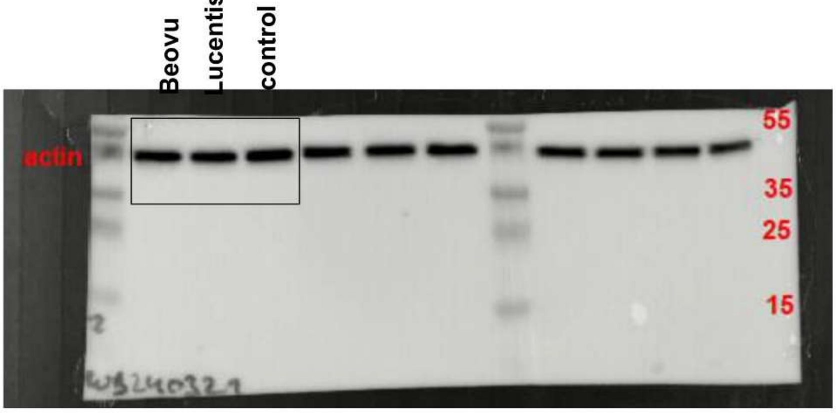

4. Lower part of the membrane was again washed in Restore Plus Western Blot Stripping Buffer at RT for 45 min to remove bound antibodies. After blocking, it was exposed to CD9-specific antibodies:

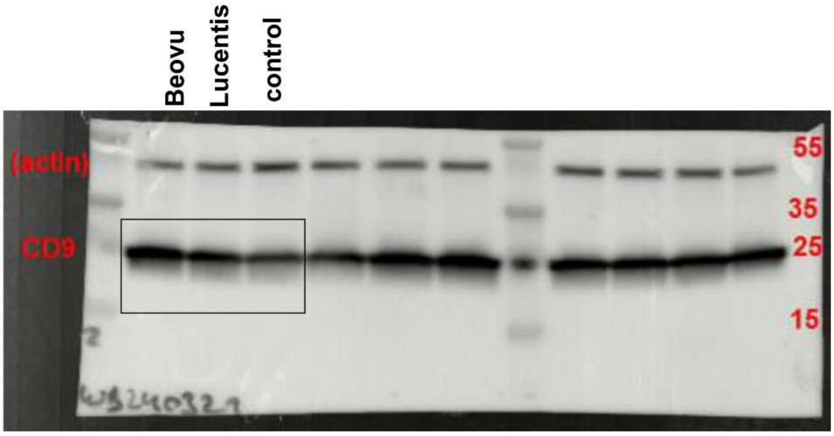

1. Cell extracts were prepared from iBREC exposed to Beovu or Lucentis for 8 d.
2. Proteins were separated by SDS-PAGE under non-reducing conditions. After protein transfer, the membrane was cut into two halves just below 70 kDa (see arrow). After blocking, the upper part was exposed to antibodies against CD29, the lower part against VEGFA-specific antibodies:

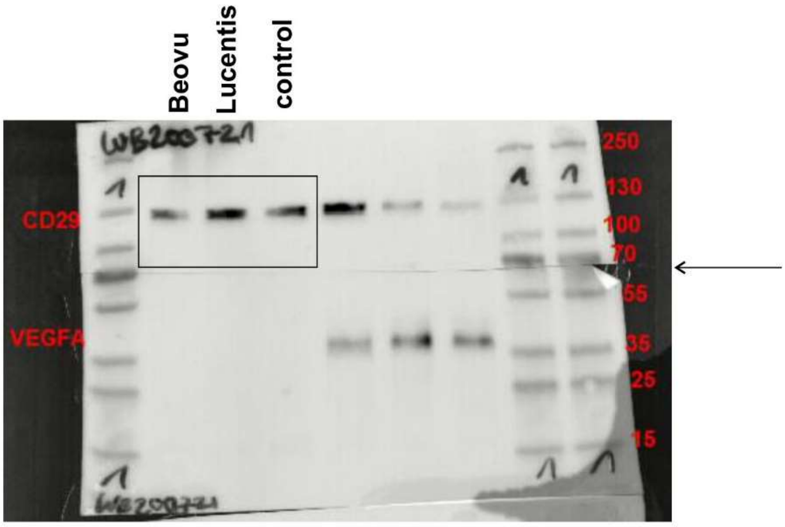

3. Lower part of the membrane was washed in Restore Plus Western Blot Stripping Buffer at RT for 45 min to remove bound antibodies before it was cut into two halves just below 35 kDa (see arrow). After blocking the upper part (35-55 kDa) was exposed to antibodies against actin, the lower part (15-35 kDa) to CD9-specific antibodies:

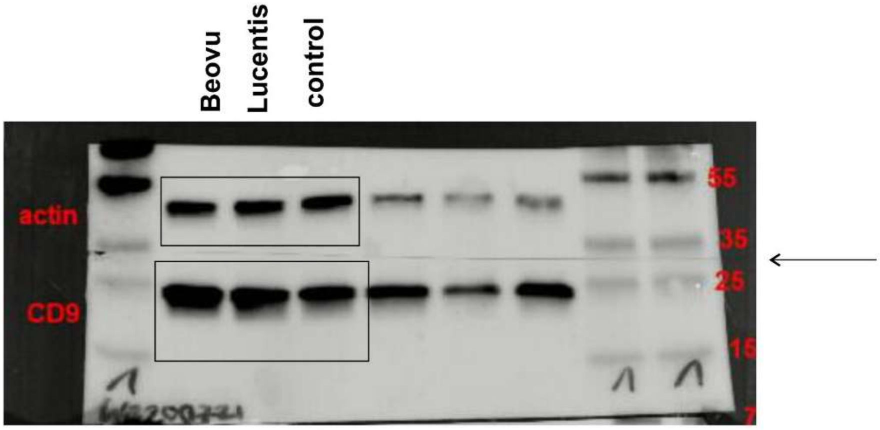

Supplementary Figure S2: Original images of Fig. 5

1. Cell extracts were prepared of iBREC exposed to 0.0002% or 0.002% polysorbate-80 for 2 d.
2. Proteins were separated by SDS-PAGE under reducing conditions. After protein transfer, the membrane was cut into two halves just between 100 kDa and 70 kDa. After blocking, the upper half was exposed to antibodies against VE cadherin, the lower half to claudin-1-specific antibodies:

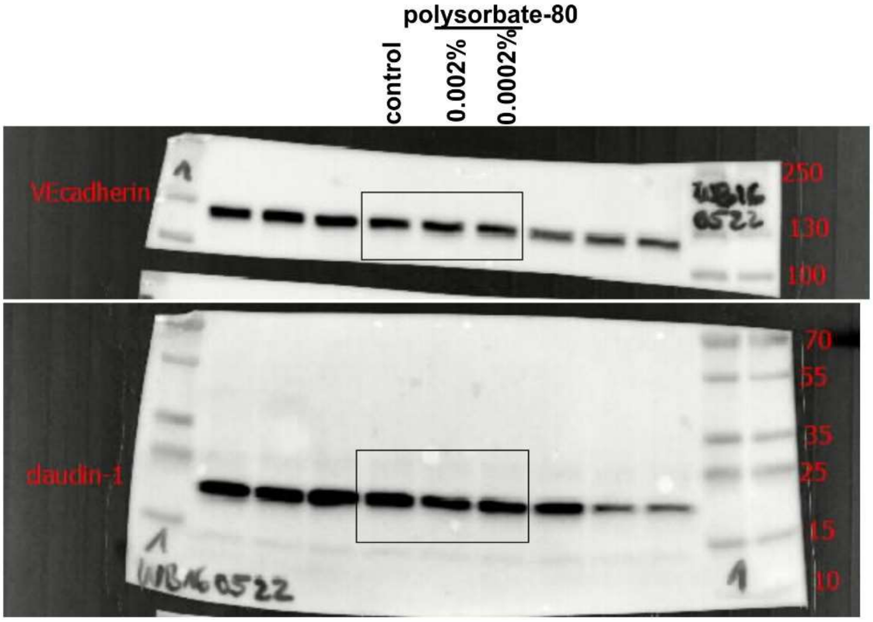

3. Lower part of the membrane was washed in Restore Plus Western Blot Stripping Buffer at RT for 45 min to remove bound antibodies. After blocking, it was exposed to actin-specific antibodies:

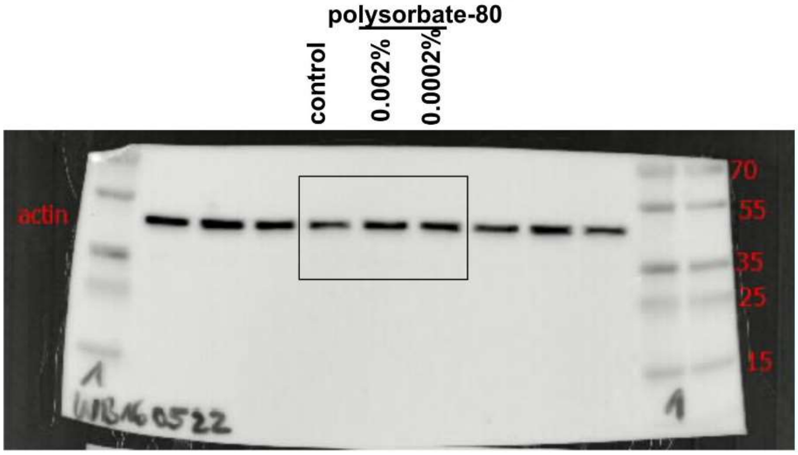

4. Lower part of the membrane was againg washed in Restore Plus Western Blot Stripping Buffer at RT for 45 min to remove bound antibodies. After blocking, it was exposed to claudin-5-specific antibodies:

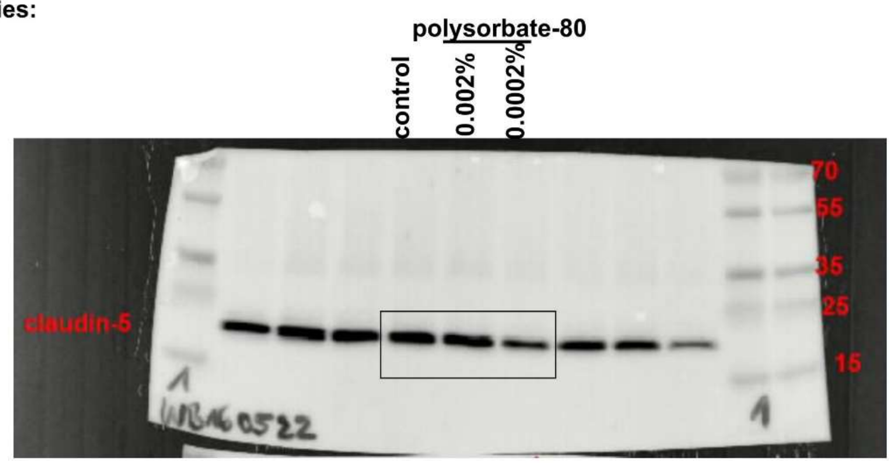

1. Cell extracts were prepared of iBREC exposed to 0.0002% or 0.002% polysorbate-20 for 2 d.
2. Proteins were separated by SDS-PAGE under reducing conditions. After protein transfer, the membrane was cut into two halves just between 100 kDa and 70 kDa. After blocking, the upper half was exposed to antibodies against VE cadherin, the lower half to claudin-1-specific antibodies:

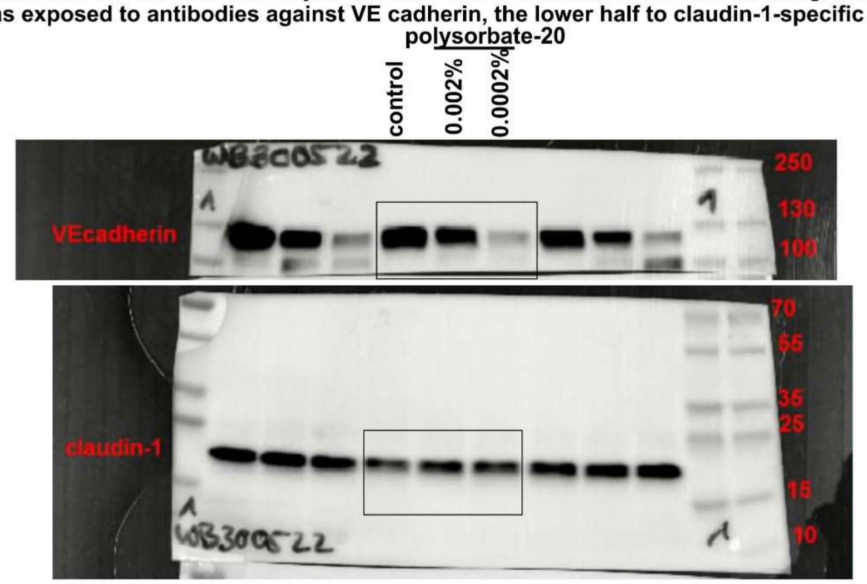

3. Lower part of the membrane was washed in Restore Plus Western Blot Stripping Buffer at RT for 45 min to remove bound antibodies. After blocking, it was exposed to actin-specific antibodies:

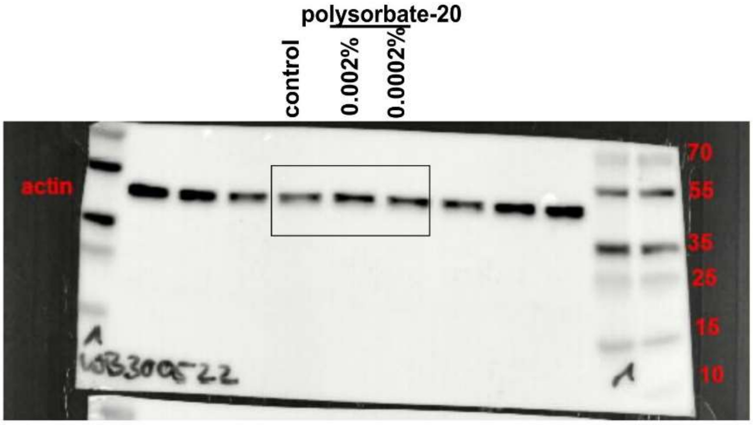

4. Lower part of the membrane was againg washed in Restore Plus Western Blot Stripping Buffer at RT for 45 min to remove bound antibodies. After blocking, it was exposed to claudin-5-specific antibodies:

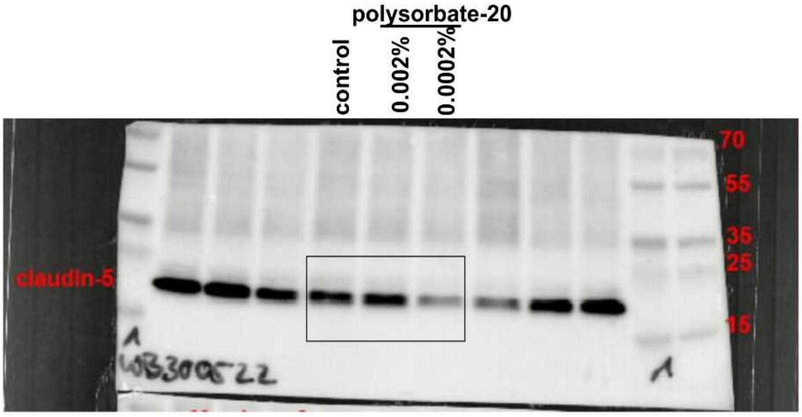

Supplement: Supplementary file 1 — Supplementary Information. [file 41598_2022_16770_MOESM1_ESM.pdf]
